# Epileptic Lunatics

**Published:** 1899-07-01

**Authors:** 


### EPILEPTIC LUNATICS.

In view of the beneficial effects which have been found to follow the employment of epileptics in open-air farm work, it seems reasonable to believe that those in whom the disease is complicated with mental deficiency will also derive advantage from a similar line of treatment. It is satisfactory, then, to learn that in connection with their new asylum on the Horton Manor estate the London County Council are about to establish a working colony for such male epileptics as are certifiable under the lunacy laws. Accommodation will be provided for about 300 such patients, who will be distributed among several houses, and kept entirely apart from the ordinary lunatics. The estate is situated in an open position on the breezy Downs near Epsom, and the portion which it is proposed to utilise for the farm colony covers 127 acres.

